# The emergence of chloroquine-sensitive *Plasmodium falciparum* is influenced by selected communities in some parts of the Central Region of Ghana

**DOI:** 10.1186/s12936-021-03985-8

**Published:** 2021-11-25

**Authors:** Kwame Kumi Asare, Justice Africa, Jennifer Mbata, Yeboah Kwaku Opoku

**Affiliations:** 1 Department of Biomedical Sciences, School of Allied Health Sciences, College of Health and Allied Sciences, University of Cape Coast, Cape Coast, Ghana; 2 Department of Medical Laboratory Science, University of Cape Coast, Cape Coast, Ghana; 3 Department of Biology Education, Faculty of Science Education, University of Education, Winneba, Ghana

**Keywords:** *Plasmodium falciparum*, Chloroquine-resistance parasites, Chloroquine-sensitive parasites, *Pfcrt*, *Pfmdr1*, Central Region, Ghana

## Abstract

**Background:**

The return of chloroquine-sensitive *Plasmodium falciparum* in sub-Saharan Africa countries offers the opportunity for the reintroduction of chloroquine (CQ) either in combination with other drugs or as a single therapy for the management of malaria. This study assesses the influence of individual study sites on the selection of CQ sensitive *P. falciparum* markers in the Central region of Ghana.

**Methods:**

Genomic DNA was extracted from an archived filter paper blood blot from Cape Coast, Elmina, Assin Fosu, and Twifo Praso using the Chelex DNA extraction method. The age metadata of the patients from whom the blood spots were taken was collected. The prevalence of CQ-sensitive markers of *pfcrt* K76 and *pfmdr1* N86 was performed using nested PCR and RFLP. The data were analysed using Chi-square and Odd ratio.

**Results:**

The overall prevalence of CQ-sensitive *P. falciparum* markers, *pfcrt* K76 and *pfmdr1* N86 in the Central Region of Ghana were 142 out of 184 (77.17%) and 180 out of 184 (97.83%), respectively. The distribution of *pfcrt* K76 was assessed among the age groups per the individual study sites. 12 out of 33 (36.36%), 8 out of 33 (24.24%) and 6 out of 33 (18.18%) of *pfcrt* K76 CQ-sensitive marker were isolated from age 0 to 5 years, 16 to 30 years and 31 to 45 years old respectively at Cape Coast. Assin Fosu and Twifo Praso had the highest *pfcrt* K76 prevalence in 0–5 years, followed by 16–30 years and 6–15 years of age. The results showed that there was a significant prevalence of *pfcrt* K76 in all study sites; Cape Coast (χ^2^ = 26.48, p < 0.0001), Assin Fosu (χ^2^ = 37.67, p < 0.0001), Twifo Praso (χ^2^ = 32.25, p < 0.0001) and Elmina (χ^2^ = 17.88, p < 0.0001). Again, the likelihood to detect *pfcrt* K76 (OR (95% CI) was 7.105 (3.118–17.14), p < 0.0001 and *pfmdr1* (2.028 (1.065–3.790), p < 0.001) among *P. falciparum* isolates from Cape Coast to be seven times and two times, respectively.

**Conclusion:**

The study showed a significant selection and expansion of chloroquine-sensitive *P. falciparum* markers in all the selected study areas in the Central region. This finding has a significant implication for the future treatment, management, and control of *P. falciparum* malaria.

**Supplementary Information:**

The online version contains supplementary material available at 10.1186/s12936-021-03985-8.

## Background

The return of chloroquine (CQ) sensitive *Plasmodium falciparum* in sub-Saharan Africa is associated with increased prevalence of wildtype *pfcrt* and *pfmdr1* variants [1, 2]. Until the emergence of *P. falciparum* resistance to CQ and its subsequent withdrawal, CQ was widely used for self-medication for suspected malaria cases [3]. CQ has resistance hampered malaria control and contributed to high mortality especially among children in sub-Saharan Africa [4, 5]. The re-emergence of CQ-sensitive *P. falciparum* parasites offers the opportunity to introduce this drug either in combination therapy or as a single anti-malarial drug [6–9]. Although the reappearance of CQ-sensitive parasites has been observed in all countries, the rate of re-emergence varies from place to place [10–12]. The central region of Ghana has experienced a slow appearance of CQ-sensitive *P. falciparum* compared to other parts of the country [13, 14]. The slow appearance of CQ-sensitive *P. falciparum* in the Central region has been attributed to the continuous usage of CQ in some parts of the Central region [13]. Early CQ treatment failure and worsening malaria conditions in young children have been reported to be higher in Tanzania, Uganda, Zambia, and Ecuador compared to Ghana [15]. Malaria prevalence has remained high while malaria-related mortality has remained stable in 5–15 years old but decreased in 0–5 years of age [16]. The withdrawal of chloroquine and introduction of artemisinin-based combination therapy (ACT) has resulted in the re-emergence of CQ-sensitive markers.

The *P. falciparum pfcrt* K76T and *pfmdr1* N86Y resistant markers are well established to be responsible for chloroquine resistance globally [17]. In Ghana, the prevalence of *pfcrt* K76T and *pfmdr*1 N86Y varies from one place to another [14, 18–25]. The *pfcrt* K76T (11%) and *pfmdr1* N86Y (8.1%) respectively have been reported as the prevalence of the CQ-resistant parasite in the Greater Accra region of Ghana in 2018 [18]. In Cape Coast, *pfcrt* K76T prevalence has gradually decrease from 47 to 53.7% in 2014, 38% in 2015, 29% in 2016 and 29% in 2017 whiles *pfmdr1* N86Y prevalence were 18–36% in 2014, 18% in 2015, 5% in 2016 and 5% in 2017 [12, 14, 19]. This is good news as CQ anti-malarial drugs could be used as a stopgap to salvage the current challenges involved in the emerging artemisinin resistance. Currently, no new anti-malarial drugs or effective malaria vaccine is available for malaria treatment [23, 24]. The increasing ACT treatment failure requires the reintroduction of CQ combination be considered as an option whiles wait for new and effective anti-malarials and vaccines [25].

Artesunate-amodiaquine (ASAQ), artemether-lumefantrine (AL), and dihydroartemisinin-piperaquine (DHAP) are the first-line anti-malarial drugs for the management of uncomplicated malaria in Ghana [26]. However, the emergence of artemisinin resistance in *P. falciparum* in South-East Asia and Rwanda has led to treatment failure of ACT [27, 28]. A current survey from 2007 to 2016 using archived blood samples from Ghana had shown that *pfk13* mutations responsible for both Asian and African artemisinin resistance were prevalent in Ghanaian *P. falciparum* parasites [29]. The study also frequently identified N599Y, K607E, and V637G non-synonymous mutations in the samples [29]. This indicates that the Ghanaian *P. falciparum* parasites can develop resistance to artemisinin [29, 30]. Therefore, the return of CQ-sensitive parasites in the country is essential as CQ combinations can be reintroduced should artemisinin treatment completely fail.

The continuous usage of CQ has been associated with the slow recovery of CQ sensitive parasites in some parts of Ghana [13, 18, 19]. The previous study showed that the proscribed usage of CQ was very important for maintaining CQ resistant markers in Central Region [13]. The report of increasing CQ sensitive markers in the region is good news, however, the prevalence of these markers is relatively low in the Central region compared to other parts of Ghana. The factors influencing the variations in the selection and emergence of CQ sensitive markers in the different malaria-endemic areas are not well understood. The study, therefore, aimed to assess the effects of the various study sites parameters on the prevalence of CQ-sensitive *P. falciparum* parasites in the Central Region of Ghana.

## Methods

### Study sites

**Fig. 1:**
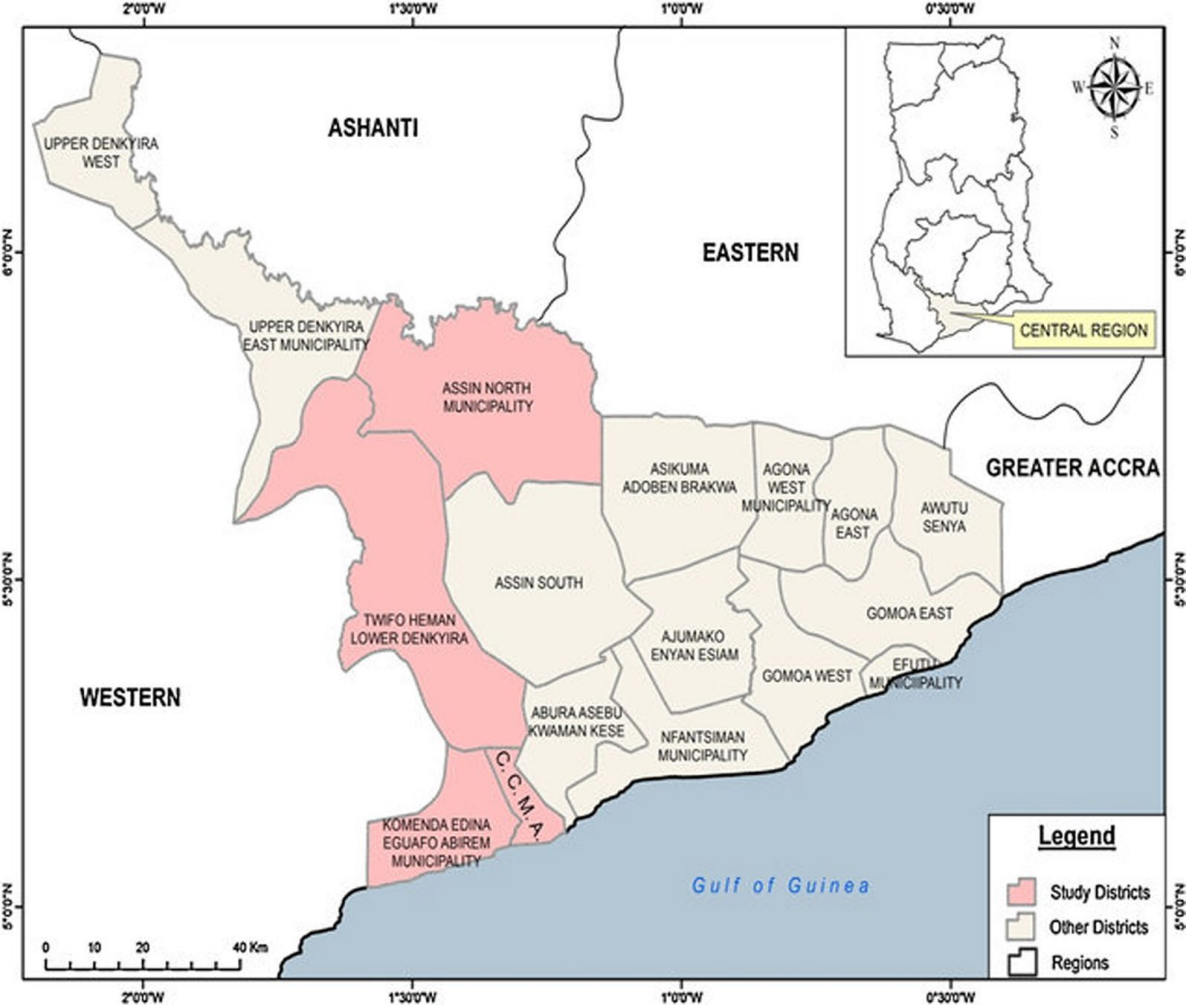
Map of the Central Region of Ghana. Map showing the district where the study was conducted

The study was conducted in four districts in the Central Region of Ghana. The samples were selected from two ecological zones with different malaria endemicity. Assin Fosu and Twifo Praso in the forest zone have a high *P. falciparum* malaria prevalence compared to Cape Coast and Elmina in the coastal zone which has lower malaria prevalence in the Central Region of Ghana. The Cape Coast metropolis which is the smallest metropolis in Ghana covers an area of 122 square km and is bounded to the West by Komenda/Edina/Eguafo/Abrem Municipality. The Gulf of Guinea is located in the southern part of the Metropolis with Abura/Asebu/Kwamankese covering the eastern boundary. It also shares its northern boundary with Twifu/Hemang/Lower Denkyira District. Cape Coast is also the Central Regional Capital of Ghana. Elmina is the district capital of the Komenda–Edina–Eguafo–Abirem (KEEA) district. The KEEA covers an area of 1372.45 square kilometres (919.95 square miles) and lies between longitude 1° 20° West and 1° 40° West and latitude 5° 05° North and 5° North 15° North. The eastern and North-Eastern bothers of KEEA are Cape Coast Metropolis and Twifo–Heman Lower Denkyira districts respectively. Twifo-Praso is located in the Twifo/Heman/Lower Denkyira district and also serves as its district capital. The total area covered by the district extends to about 1199 square kilometres and lies within latitudes 5° 50° N and 5° 51° N and longitudes 1° 50° W and 1° 10° W with about 1510 settlements. Finally, the Assin North municipality is made up of about 1000 settlements. Major among them include Assin Fosu, Assin Akonfudi, Assin Kushea, Assin Praso, etc. The Municipal lies within longitudes 1° 05° East and 1° 25° West and latitudes 6° 05° North and 6° 40° South with a land coverage of about 1500 square kilometres. It is bounded by upper Dankyira, Ajumako Enyan-Esiam, Assin South Districts as well as lower Dankyira (Fig. 1) [13, 19].

### Sample collection

The sample size, 214, was calculated using the following method as described; n = z^2^ pq/d2, where n = the desired sample size (when the population is greater than 10,000); z = the standard normal deviation, usually set at 1.96, which corresponds to the 95% confidence interval; p = the proportion in the target population estimated to have a particular characteristic(s); If there is no reasonable estimate, then 50% is used; q = 1.0 − p; d = degree of accuracy desired, usually set at 0.05 level or occasionally at 0.02. Each selected site has a population greater than 10, 000. The standard deviation (z) was set at 1.96, which corresponds to the 95% confidence level. The *pfcrt* K76 prevalence among malaria isolates in Ghana (88.4%) [31] was used as the proportion in the target population estimated to have a particular characteristic (which, in this case, is malaria) (p). Therefore p = 0.884, and q = 1 – 0.884 = 0.116. The degree of accuracy was set at 0.05. Hence n = z^2^ pq/d2 n = (1.962) (0.884) (0.116)/(0.052), n = 0.39393/0.0025, n = 157.57. However, 214 *P. falciparum* parasites were analysed from the four study sites [19, 20].

Archived *P. falciparum-*infected blood blot samples (214) previously successful extracted from Cape Coast, Elmina, Twifo Praso, and Assin Fosu collected between April 2012 and December 2013 were used for this study [19, 31]. The air-dried blood spots in zip-locked plastic envelopes containing silica gel stored at − 20 °C freezer in the Department of Biomedical Sciences, University of Cape Coast were extracted using the chelex extraction method [19, 31]. The excel data which contains the sample numbers and patient information at the Department were obtained for the analysis (Additional file 1: Fig. 1).

### Genomic DNA extraction

The Chelex-saponin DNA extraction method was used [13]. Briefly, discs punched of about 2.5 mm (2×) were made from the dried blood spot then transferred into a 1.5 mL Eppendorf tube. 50 µL of 10% saponin and 1 mL of 1× PBS were added to the blood spots in 1.5 mL tubes. The sample and saponin mixture was vortexed and frozen at 4 °C overnight. The filter was washed 3× with 1 mL of 1× PBS, followed by 30 µL of 20% chelex and 70 µL of DNase/RNase water. The sample was incubated at 95 °C for 10 min with intermittent vortexing. The genomic DNA (gDNA) samples were eluted from the filter by centrifuging at 13,000 rpm for 6 min and the supernatant was transferred into a sterile 0.5 mL microfuge tube. The extracted gDNA was stored at − 20 °C. [13, 19, 31]

### Amplification of *pfcrt *and *pfmdr1* by nested polymerase chain reaction (PCR)

The genomic DNA of *P. falciparum* was amplified using primer pairs (P10-1forw: TTGTCGACCTTAACAGATGGCTCAC/P10-1 rev: AATTTCCCTTTTTATTTCCAAATAAGGA for *pfcrt* & P1-1 forw: TTAAATGTTTACCTGCACAACATAGAAATT/P1-1 rev: CTCCACAATAACTTGCAACAGTTCTTA for mdr1, primary reaction and P10 forw: CTTGTCTTGGTAAATGTGCTC/P10 rev: GAACATAATCATACAAATAAAGT for *pfcrt* & P1 forw: TGTATGTGCTGTATTATCAGGA/P1 rev: CTCTTCTATAATGGACATGGTA for secondary reaction). The primary PCR reaction mixture contained 0.2 µM of the primary primer pair, 5 μL DNA template, 1× PCR buffer, 200 µM dNTPs, 1.5 mM MgCl_2_, and 1.25 U of *Taq* DNA Polymerase in a 25 μL mixture. The PCR cycling conditions (95 °C for the 30 s, [95 °C for 15 s, 53 °C for 1 min, 68 °C for 1 min], 68 °C for 5 min final extension). The secondary PCR was performed using 0.5 μL of the primary PCR reaction and 133.33 nM each of the second primer pairs. The PCR cycling condition was the same except for the annealing temperature which was 60 °C for the second PCR. The secondary PCR products (1 µL) were run on a 2% agarose gel and visualized using a CSL gel documentation system (CSL, UK) (Additional file 2).

### Restriction fragment length polymorphism (RFLP) [13, 32]

The nested PCR products of *pfcrt* were digested with Apo I whereas that of *Pfmdr1* were digested with Apo I and Afl III. The digested products (5 µL) were separated on 2% electrophoresis gel, stained with ethidium bromide, and viewed under ultraviolet light. The *pfcrt* PCR product contains a single Apo I site if the codon 76 of the *pfcrt* gene encodes for lysine (K76). For *pfmdr1*, codon 86 was digested with both Apo I for asparagine and Afl III to confirm the tyrosine mutation at position 86 in all the *pfmdr1* samples that were not digested by Apo I. The digested samples were run on 2% agarose gel and visualized using a CSL gel documentation system.

### Data analysis

The data from RFLP analysis, patient ages & sample collection sites were organized using Microsoft Office Excel 2010 (Microsoft Corporation) and the statistical analyses were performed with GraphPad Prism software, version 8.4.3 (GraphPad Software). All mixed alleles infection of wild type and resistant strains of *pfcrt* K76T and *pfmdr1* N86Y (Cape coast: n = 6/46 (13.04%), Elmina: n = 8/39 (20.15%), Twifo Praso: n = 8/60 (13.33%) and Assin Fosu n = 8/69 (11.59%)) were excluded from the analysis because these could not be classified as solely resistant or CQ-sensitive parasites. The ages of the patients were categorized into 0–5 years, 6–15 years, 16–30 years, 31–45 years & above 45 years for the analysis. The statistical significance was determined using 2 × 2 contingency Chi-square and odds ratio. A p-value < 0.05 was considered statistical significance.

## Results

**Fig. 2:**
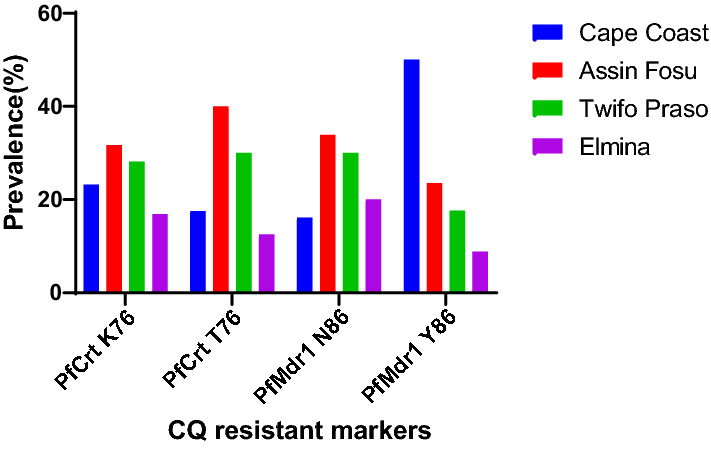
The prevalence of chloroquine sensitive and resistance markers *pfcrt* K76T and *pfmdr1* N86Y at the study sites

The overall prevalence of CQ-sensitive *P. falciparum* markers, *pfcrt* K76 and *pfmdr1* N86 in the Central Region of Ghana were 142 out of 184 (77.17%) and 180 out of 184 (97.83%), respectively. Assin Fosu harboured the highest number of *P. falciparum* expressing CQ-sensitive *pfcrt* K76 marker 45 out of 142 (31.69%) followed by Twifo Praso 40 out of 142 (28.17%) samples analysed. A similar observation was made for the *pfmdr1* N86 CQ-sensitive gene (Fig. 2). 33 (82.5%), 45 (73.77%), 40 (76.92%) and 24 (97.83%) were the prevalences of *pfcrt* K76 and 29 (63.04%), 61 (88.40%), 54 (90.00%) and 36 (92.30%) were the prevalence of *pfmdr1* N86 at Cape Coast, Assin Fosu, Twifo Praso and Elmina study sites, respectively (Table 1).

**Table 1:** Prevalence of *pfcrt* K76T and *pfmdr1* N86Y at the study sites

Study sites	Pfcrt 76 n/N(%)		Pfmdr1 86 n/N (%)	
	K	T	N	Y
Cape Coast	33/40 (82.5%)	7/40 (17.5%)	29/46 (63.04%)	17/46 (36.96%)
Assin Fosu	45/61 (73.77%)	16/61 (26.23%)	61/69 (88.40%)	8/69 (11.60%)
Twifo Praso	40/52 (76.92%)	12/52 (23.08%)	54/60 (90.00%)	6/60 (10.00%)
Elmina	24/29 (82.76%)	5/29 (17.24%)	36/39 (92.3%)	3/39 (7.69%)

**Fig. 3:**
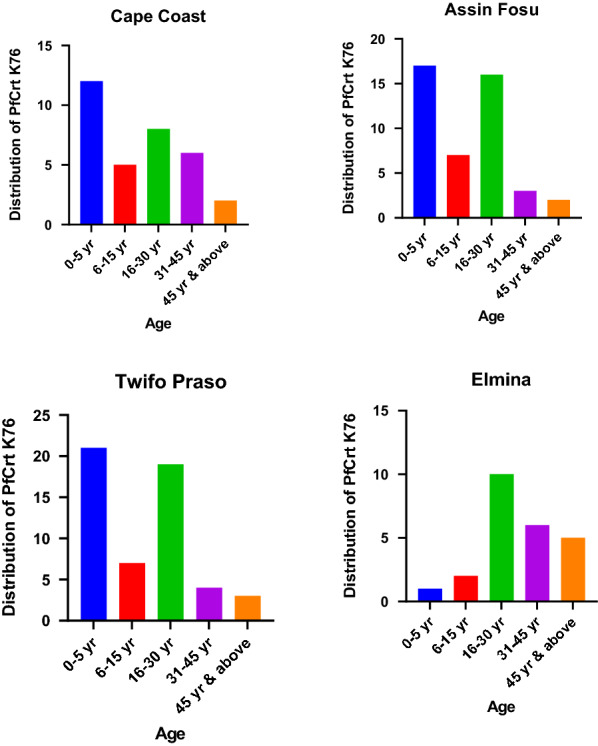
The distribution of chloroquine of *pfcrt* K76 chloroquine-sensitive marker among the age groups at the study sites in the Central Region

**Fig. 4:**
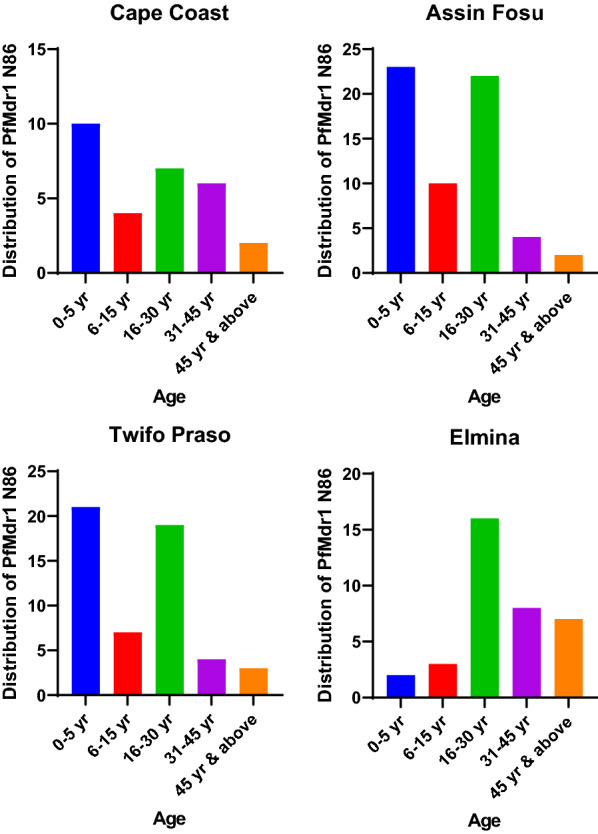
The distribution of chloroquine of *pfmdr1* N86 chloroquine-sensitive marker among the age groups at the study sites in the Central Region

The distribution of *pfcrt* K76 was assessed among the age groups for the individual study sites. 12 out of 33 (36.36%), 8 out of 33 (24.24%) and 6 out of 33 (18.18%) of *pfcrt* K76 CQ-sensitive markers were isolated from age 0–5 years, 16–30 years and 31–45 years old respectively at Cape Coast. Assin Fosu and Twifo Praso had the highest *pfcrt* K76 prevalence in 0–5 years, followed by 16–30 years and 6–15 years of age. However, the highest prevalence of *pfcrt* K76 was observed among subjects between the age group 16–30 years, followed by 31–45 years and above 45 years of age at the Elmina study site (Fig. 3). Similar observations were made for *pfmdr1* N86 CQ-sensitive markers at each of the individual study sites with Assin Fosu having the highest *pfmdr1* prevalence in 23 out of 61 (37.70%) in 0–5 years followed by 22 out of 61 (36.07%) in 16–30 years of age (Fig. 4).

To determine the influence of study sites on the prevalence of CQ-sensitive markers *pfcrt* K76 and *pfmdr1* N86, an association study was conducted. The result should that there is a significant prevalence of *pfcrt* K76 in all study sites; Cape Coast (χ^2^ = 26.48, p < 0.0001), Assin Fosu (χ^2^ = 37.67, p < 0.0001), Twifo Praso (χ^2^ = 32.25, p < 0.0001) and Elmina (χ^2^ = 17.88, p < 0.0001). Similar observations were made for the prevalence of *pfmdr1* N86 CQ-resistant marker at Cape Coast (χ^2^ = 4.977, p = 0.0257), Assin Fosu (χ^2^ = 42.94, p < 0.0001), Twifo Praso (χ^2^ = 51.75, p < 0.0001) and Elmina (χ^2^ = 30.85, p < 0.0001) (Table 2).

**Table 2:** Association the study sites and the prevalence of prevalence of Chloroquine sensitive markers

Town	Pfcrt K76			Pfmdr1 N86		
	χ^2^	z-test	p	χ^2^	z-test	p
Cape coast	26.48	5.145	< 0.0001	4.977	2.231	0.0257
Assin Fosu	37.67	6.138	< 0.0001	42.94	6.553	< 0.0001
Twifo Praso	32.25	5.679	< 0.0001	51.75	7.194	< 0.0001
Elmina	17.88	4.228	< 0.0001	30.85	5.555	< 0.0001

The odds of detecting *P. falciparum* isolates harbouring CQ-sensitive *pfcrt* K76 and *pfmdr1* N86 at each of the study sites were assessed. The results showed that there the odds for the detection of *pfcrt* K76 (OR (95% CI) was 7.105 (3.118–17.14), p < 0.0001 and that of *pfmdr1*was 2 (2.028 (1.065–3.790), p < 0.001) among *P. falciparum* isolates from Cape Coast. Assin Fosu had the highest likelihood for the detection of *pfcrt* K76, OR (95% CI) 8.477 (3.926–17.83), p < 0.0001 and Elmina had the highest odd of the detection of *pfmdr1* N86 OR (95% CI) 14.27 (4.795–44.67), p < 0.0001 among the *P. falciparum* isolates in Central Region of Ghana (Table 3).

**Table 3:** The odds of detecting chloroquine-sensitive markers (*pfcrt* K76 and *pfmdr1* N86) among *P. falciparum* isolate at the study sites

Town	Pfcrt K76		Pfmdr1 N86	
	Odd ratio (95% CI)	p	Odd ratio (95% CI)	p
Cape coast	7.105 (3.118–17.14)	< 0.0001	2.028 (1.065–3.790)	< 0.001
Assin Fosu	8.477 (3.926–17.83)	< 0.0001	9.065 (4.206–18.96)	< 0.0001
Twifo Praso	7.535 (3.425–15.89)	< 0.0001	13.56 (5.972–29.93)	< 0.0001
Elmina	5.177 (2.367–11.15)	< 0.0001	14.27 (4.795–44.67)	< 0.0001

## Discussion

The recovery of the CQ-sensitive *P. falciparum* has come at a time when the treatment and management of malaria are constrained with the development of resistance to almost all the most effective anti-malarial drugs [32, 33]. Although the current advances and knowledge in malaria biology have the potential to identify drug targets for the development of safe, effective, and efficient anti-malarial drugs and vaccines for the management of malaria, they are yet to produce an effective means to tackle the challenges facing malaria treatment and management [34, 35]. As exploration for the new treatment strategies continues coupled with the identification of novel drug targets and the design of new anti-malarial compounds, it is important to understand the dynamism and factors that influence the re-emergence of CQ-sensitive *P. falciparum* in malaria-endemic areas [36, 37]. This will better position scientists in the implementation of appropriate protocols for the re-introduction of CQ as a temporal strategy to control malaria as we wait for the new effective and safe anti-malarial drugs and vaccines [38]. This study focuses on assessing the effects of study sites on the prevalence and the emergence of CQ-sensitive markers, *pfcrt* K76 and *pfmdr1* N86 alleles among *P. falciparum* isolate in the Central region of Ghana.

The study showed a reduction in CQ-resistant markers in the Central region of Ghana. A similar result has been reported in other parts of Ghana [12]. Although the steady decline of *pfcrt* T76 and *pfmdr1* Y86 has been very slow from 62 to 89% between 2014 and 2017 especially at the regional capital (Cape Coast) of the Central region of Ghana compared to other places in Ghana, the expansion of the CQ-sensitive *P. falciparum* strains have increased appreciably [14, 19, 31]. Similar reports on the reappearance of CQ-sensitive parasites have been reported in Malawi, Cameroon, Ethiopia, Nigeria, Tanzania, and Mozambique [6, 10, 11, 39–42]. The decrease in CQ-resistant markers and subsequent parasite sensitivity to CQ treatment have been demonstrated clinically [43]. The slow appearance of *pfcrt* K76 & *pfmdr1* N86 suggests that the maintenance of the integrity of CQ-resistant phenotypes has fitness costs in the absence of CQ drug pressure [44, 45]. The absence of CQ drug pressure results in the negative selection of CQ-resistant parasites; thus, driving the evolution of CQ-sensitive parasites [45]. Thus, the reappearance of CQ-sensitive *pfcrt* K76 & *pfmdr1* N86 alleles has also been observed in malaria transmission.

Also, the recovery of CQ-sensitive parasites is determined by eco-epidemiological factors such as migration and vector competence within an area. Thus, the expansion of *pfcrt* K76 & *pfmdr1* N86 observed among the study sites could be attributed to migration. A previous report indicated the possibility of *pfcrt* sensitive parasites either upsurging from low levels in the indigenous parasite populations or migrating from bordering countries with persistent CQ drug pressure [46]. This observation supports the idea of migration of CQ-sensitive phenotype from places with persistent CQ pressure [1]. All the study sites were significantly associated with the prevalence of *pfcrt* K76 and *pfmdr1* N86. The odds of detecting CQ-sensitive markers, *pfcrt* K76 was high at Assin Fosu, Twifo Praso and Cape Coast whereas odds of detecting CQ-sensitive markers, *pfmdr1* N86 was high at Elmina, Twifo Praso and Assin Fosu. Such migration of *pfcrt* K76 & *pfmdr1* N86 alleles could be due to rapid movements of goods and services as well as human traffic among Cape Coast (the regional capital), Assin Fosu (Assin north municipal capital), and Twifo Praso (the district capital of Twifo Heman Lower Denkyira) cyclically. It has also been shown that transiting from malaria-endemic areas to hypoendemic areas exposes people to the changing risks of malaria infections [47, 48]. Thus, the pattern of malaria infection within a community is significantly influenced by the movement of people [49]. The association of the prevalence of *pfcrt* K76 & *pfmdr1* N86 among the study areas take a similar pattern of the spread of drug resistance lineage parasites [50]. Thus, the continuous usage or complete withdrawal of CQ from an area determines the level of prevalence of CQ drug-resistant parasites within the area.

## Limitations

The study used archived samples and therefore could not allow for assessment of critical factors such as immunity and migration which are very important in the evolution of drug-resistant alleles. The prevalence of drug-resistant *P. falciparum* are changing which require large quantities of field samples and the assessment of host factors, parasite factors and environmental factors need to be constantly updated to provide the current status of anti-malarial drug resistance in the Central Region of Ghana.

## Conclusion

The study has shown an association between study sites and the prevalence of CQ-sensitive markers with high odds for detecting *pfcrt* K76 and *pfmdr1* N86 among *P. falciparum* isolates in the Central Region of Ghana. These findings have significant implications for the future treatment, management, and control of *P. falciparum* malaria*.*

## Supplementary Information

**Additional file 1: Figure 1.** Restriction fragment length polymorphism for *pfcrt* 76 and *pfmdr1* 86 alleles. (1) RFLP of *pfcrt* 76: Apo I digestion of *pfcrt* amplicons containing codon 76 polymorphism. Lane M is 100–1000 bp molecular size marker, lane 1 is a known sample, and lanes 2–21 are *P. falciparum* isolates. The wild-type allele has amplicons 89 bp and 123 bp whiles the mutant-type allele has 200 bp and mixed infection has three amplicons. (2) RFLP of *pfmdr1* 86: Afl III digestion of *pfmdr1* amplicons containing codon 86 polymorphism. Lane M is 100–1000 bp molecular size marker, lane 1 is a known sample, and lanes 2–21 are *P. falciparum* isolates. The mutant-type allele has two amplicons 200 bp and 300 bp amplicons compared to the wildtype with 240 bp and 260 bp.

**Additional file 2.** Sections of the data analysed and a sample of Chi square and odd ratio analysis for pfcrt K76T at Cape Coast.

## Data Availability

Not applicable.
