# Supplementary figures and images for: The emergence of chloroquine-sensitive Plasmodium falciparum is influenced by selected communities in some parts of the Central Region of Ghana
Source: Malar J. 2021 Nov 25;20:447. doi: 10.1186/s12936-021-03985-8 (PMC8620919; doi:10.1186/s12936-021-03985-8)

## Slide 1
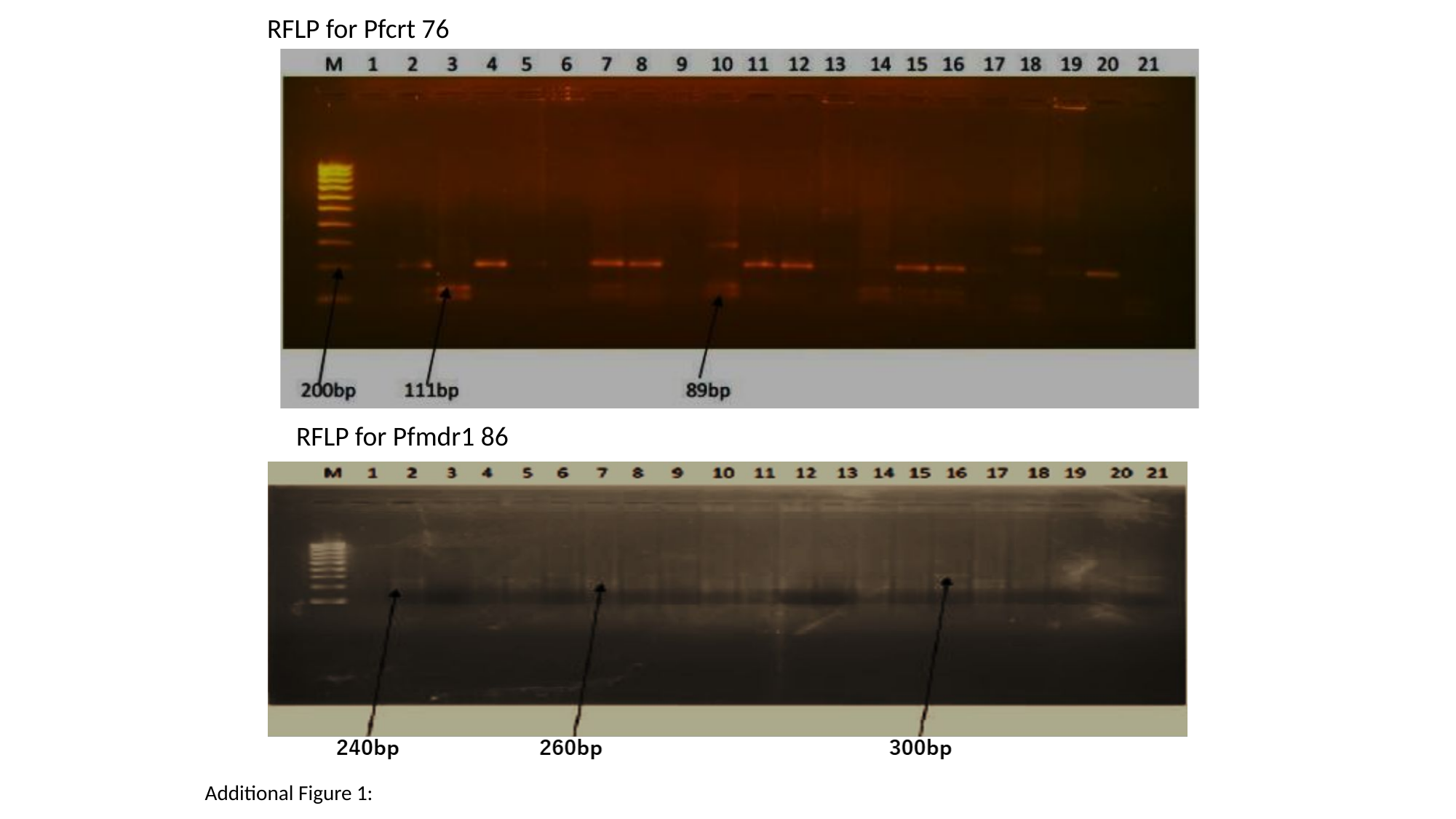

RFLP for Pfcrt 76
RFLP for Pfmdr1 86
Additional Figure 1:

Supplement: Supplementary file 1 — Additional file 1: Figure 1. Restriction fragment length polymorphism for pfcrt 76 and pfmdr1 86 alleles. (1) RFLP of pfcrt 76: Apo I digestion of pfcrt amplicons containing codon 76 polymorphism. Lane M is 100–1000 bp molecular size marker, lane 1 is a known sample, and lanes 2–21 are P. falciparum isolates. The wild-type allele has amplicons 89 bp and 123 bp whiles the mutant-type allele has 200 bp and mixed infection has three amplicons. (2) RFLP of pfmdr1 86: Afl III digestion of pfmdr1 amplicons containing codon 86 polymorphism. Lane M is 100–1000 bp molecular size marker, lane 1 is a known sample, and lanes 2–21 are P. falciparum isolates. The mutant-type allele has two amplicons 200 bp and 300 bp amplicons compared to the wildtype with 240 bp and 260 bp. [file 12936_2021_3985_MOESM1_ESM.pptx]
